# Time trends in incidence, comorbidities and socio-demographic risk factors for diagnosed autism spectrum disorder among children and adolescents in Finland

**DOI:** 10.1192/j.eurpsy.2025.374

**Published:** 2025-08-26

**Authors:** Z. Kafami, S. Upadhyaya, T. Ståhlberg, A. Sourander

**Affiliations:** 1 Research center for child psychiatry; 2 INVEST Research Flagship Center, Turku University, Turku, Finland

## Abstract

**Introduction:**

ASD is a neurodevelopmental disorder with 1% prevalence among children and adolescents, with high comorbidity with Intellectual Disability. ASD imposes a significant disease burden and is a leading cause of disability among children and adolescents. Its aetiology is multifactorial, including both genetic and environmental factors. Although prior studies have explored the incidence, comorbidities and several prenatal risk factors, research especifically focusing on ASD categorization by ID is limited.

**Objectives:**

The aim of this study was to report the incidence and cumulative incidence of overall ASD, and HF and LF-ASD categorization, utilizing several national registers.

**Methods:**

This study consisted of all singltone born in Finland between 1998 and 2015 (N=1,044,102), who were diagnosed with ASD from age 3 to 20. We devided study sample into four cohorts by birth year: 1998-2002, 2003-2007, 2008-2011 and 2012-2015. We also categorized ASD into LF-ASD and HF-ASD, based on their comorbidity with ID (ICD 10 codes: F70-F79). All cases (n=10,171) were matched with Controls (n=49,391) on the age, gender and place of birth. We limited our study of other psychiatric disorders comorbidities to the oldest birth cohort, born in 1998-2002. The association between sociodemographic risk factors and ASD cases and separately for HF-ASD and LF-ASD was analysed using Conditional logistic regression.

**Results:**

The incidence of ASD increased with age, specifically among girls. Among the birth cohorts, the cumulative incidence of HF-ASD increased from 0.52 (95% CI 0.49-0.54) to 0.89 (95% CI 0.84- 0.94), by age 10, while in the cumulative incidence of LF-ASD remained stable at 0.17 (95% CI 0.16-0.19). Several socio-demographic risk factors were associated with both HF-ASD and LF-ASD, except for parental immigration, which was only associated with LF-ASD. A total of 58.99% cases had at least one comorbid diagnosis, 35.91% of HF-ASD and 31.86% of LF-ASD. The most common comorbid diagnoses among HF-ASD cases were ADHD (33.9%), depressive disorder (25.4%) and anxiety disorder (23.9%). Among LF-ASD cases were ADHD (17.2%), anxiety disorder (10.5%) and Schizophrenia (5.2%).

**Conclusions:**

The incidence of diagnosed ASD increased with age from 1998-2018, especifically HF-ASD. This result could indicate a real increase in incidence or is due to improved access to mental health services and enhanced awareness of ASD among population and professionals. The rise in ASD incidence underscores the need for specialized mental health services and social support. The association between immigration and the risk of ASD highlights the importance of ensuring mental health services are accessible to immigrants.

**Disclosure of Interest:**

None Declared

